# Flexible Three-Dimensional Force Tactile Sensor Based on Velostat Piezoresistive Films

**DOI:** 10.3390/mi15040486

**Published:** 2024-03-31

**Authors:** Yuanxiang Zhang, Jiantao Zeng, Yong Wang, Guoquan Jiang

**Affiliations:** 1 College of Mechanical Engineering, Quzhou University, Quzhou 324000, China; 221122020350@zjut.edu.cn (J.Z.); wangy@zjut.edu.cn (Y.W.); jgq@zjut.edu.cn (G.J.); 2 College of Mechanical Engineering, Zhejiang University of Technology, Hangzhou 310023, China

**Keywords:** flexible sensor, three-dimensional force sensor, velostat, piezoresistive

## Abstract

The development of a high-performance, low-cost, and simply fabricated flexible three-dimensional (3D) force sensor is essential for the future development of electronic skins suitable for the detection of normal and shear forces for several human motions. In this study, a sandwich-structured flexible 3D force tactile sensor based on a polyethylene-carbon composite material (velostat) is presented. The sensor has a large measuring range, namely, 0–12 N in the direction of the normal force and 0–2.6 N in the direction of the shear force. For normal forces, the sensitivity is 0.775 N^−1^ at 0–1 N, 0.107 N^−1^ between 1 and 3 N, and 0.003 N^−1^ at 3 N and above. For shear forces, the measured sensitivity is 0.122 and 0.12 N^−1^ in *x*- and *y*-directions, respectively. Additionally, the sensor exhibits good repeatability and stability after 2500 cycles of loading and releasing. The response and recovery times of the sensor are as fast as 40 and 80 ms, respectively. Furthermore, we prepared a glove-like sensor array. When grasping the object using the tactile glove, the information about the force applied to the sensing unit can be transmitted through a wireless system in real-time and displayed on a personal computer (PC). The prepared flexible 3D force sensor shows broad application prospects in the field of smart wearable devices.

## 1. Introduction

Human skin is a complex sensing organ that can perceive various physical stimuli and plays an important role in the interactions between humans and the external environment. With the rapid development of intelligent sensing technology and functional materials, electronic skin (e-skin) is widely used in the fields of health monitoring [1,2], smart robotics [3,4,5,6], and wearable devices [7,8], etc. As an important component of e-skin, the flexible pressure sensor is gradually becoming a research hotspot. However, traditional single-axis pressure sensors are unable to meet the demands of complex, advanced, intelligent systems, while flexible 3D force sensors with unique multidirectional-mechanical detection capabilities have gained widespread attention. Compared with conventional pressure sensors, flexible 3D force tactile sensors can not only simultaneously detect normal and shear forces but also have other advantages, such as excellent elasticity, high sensitivity, and simple fabrication processes. Flexible 3D force sensors are capable of detecting comprehensive and accurate mechanical information in 3D space [9,10,11]. Thus, they can be used for detecting the texture and slip of objects [12,13].

Generally, the inherent properties of the material and the structure of flexible 3D force sensors are important factors that affect their performance. Several high-performance nanomaterials, including carbon nanotubes (CNTs) [14], carbon black (CB) [15], graphene oxide (GO) [16], and metal nanoparticles, have been employed as sensitive materials. In addition, polydimethylsiloxane (PDMS), polyurethane (PU), and silicone rubber (SR) exhibit excellent properties, such as exceptional electrical insulation, cflexibility, and biocompatibility, which enable them to be widely used as flexible substrates for wearable electronics [15,16,17,18,19]. Although conductive nanomaterials and flexible substrates provide an important foundation for the preparation of flexible 3D force tactile sensors, the manufacturing of sensors exhibiting high sensitivity, excellent cycling stability, and fast response based on simple and low-cost preparation processes still faces huge challenges. Based on their sensing mechanism, flexible 3D force sensors can be classified into piezoresistive [20,21,22], piezoelectric [23,24,25], capacitive [26,27,28], triboelectric [29,30,31], and optical [32] sensors, which all generate different types of signals under mechanical stimuli, such as pressure, stretching, bending, and torsion. Compared with the common capacitive and piezoelectric mechanisms, piezoresistive sensors exhibit a higher sensitivity, faster dynamic response, and larger measurement range. Additionally, their preparation process is simple and inexpensive, making these sensors widely used in wearable electronic devices. For these reasons, piezoresistive sensors were selected for consideration in this work.

Here, we present a sandwich-structured flexible 3D force sensor that can be fabricated through a practical and low-cost multilayer assembly method. Through finite element analysis (FEA), a bump with an edge length of 7 mm with a better conduction force is selected, which can concentrate the external force in the sensing area to improve the sensitivity. The sensor has excellent characteristics in terms of measurement range, stability, and response time. In addition, a glove-like tactile-sensor array was demonstrated that provides real-time feedback on tactile force. The prepared flexible 3D force sensor and corresponding array also show significant potential for application within the field of flexible wearable devices.

## 2. The 3D Force-Sensing Mechanism

**Figure 1:**
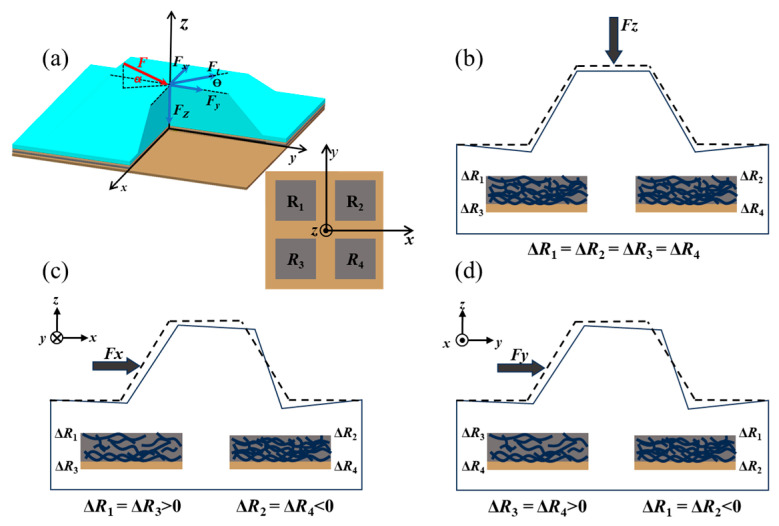
Diagram of the sensing mechanism of piezoresistive flexible 3D force sensors: (**a**) detection principle; (**b**) response to a normal force; (**c**) response to a shear force in the *x*-axis; (**d**) response to shear force in the *y*-axis.

The detection principle underlying the piezoresistive flexible 3D force sensor is illustrated in Figure 1. As shown in Figure 1a, an arbitrary force can be decomposed into three components, that is, the normal force *F_z_*, shear force *F_x_*, and *F_y_*. Additionally, *F_t_* is the resultant force of the two shear components, the angle *θ* represents the direction of the applied force in the horizontal plane, and the angle *α* represents the angle between the arbitrary force *F* and the horizontal plane.

An individual sensor is composed of four piezoresistive sensing units (namely, *R*_1_, *R*_2_, *R*_3_, and *R*_4_) distributed about the center with a four-fold symmetry. As shown in Figure 1b, under the normal force component, the bump is compressed, and the four piezoresistive sensors are subjected to the same compressive stress. Through the piezoresistive effect of the conducting material, the resistance of the four sensors decreases simultaneously, and the magnitudes of the changes are equal (Δ*R*_1_ = Δ*R*_2_ = Δ*R*_3_ = Δ*R*_4_). As shown in Figure 1c,d, the bump deforms and generates a torque at the fixed end under shear force. The two sensing units on one side of the sensor are subjected to compressive stress, while the two sensing units on the other side are subjected to tensile stress. The resistance values of the sensing units on both sides of the sensor show opposite changes. Thus, the normal and shear forces can be calculated from the average of and the difference in the values of the resistance of the four sensing units, respectively.

## 3. Design and Manufacturing of the Sensor

### 3.1. Material

The pressure-sensitive conductive material has a significant effect on the performance of piezoresistive flexible 3D force sensors. Velostat [33,34] is widely used in electronic components, flexible wearable devices, and touchscreens due to its flexibility and stability. Velostat is a polymer conductive material, which is mainly composed of polyethylene and CB particles [35]. Polyethylene is a common plastic material with corrosion resistance and good chemical stability, and CB particles provide the material with good electrical conductivity. In addition, the manufacturing process of velostat material is relatively simple, which makes its production cost low [36]. Its material properties are shown in Table 1. The resistance change in velostat is mainly caused by quantum tunneling and via the percolation phenomena [37]. When external forces are applied, the distance between the conductive CB particles in velostat will change, causing electrons to cross the potential barrier through the quantum-tunneling effect and conducting the current at a lower energy level. At the same time, the percolation phenomena will also lead to an increase in the contact area between the conductive CB particles and promote the larger transmission of the charge [38]. Thus, it significantly changes the resistivity of the material. The combined action of these two physical phenomena causes velostat to show different resistance characteristics when under pressure, making it an effective pressure-sensitive material that has important applications in pressure sensors and other fields [39]. The velostat material was supplied by the Sizhirui Technology Company Limited (Wuxi, China). PDMS and 3140 adhesives were purchased from Dow Corning, USA. Polyethylene terephthalate (PET) tape was supplied by the Baojiaxin Technology Company Limited (Shenzhen, China).

### 3.2. Structural Design

#### 3.2.1. Design of the Bump

**Figure 2:**
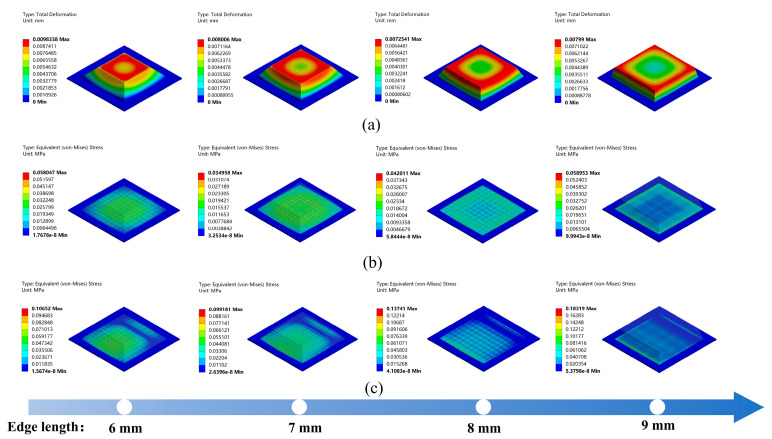
FEA results for bumps with different sizes: (**a**) bump deformation at a normal force of 1 N; (**b**) equivalent effect diagram of the piezoelectric film; and (**c**) equivalent effect diagram of the piezoresistive film at both normal and shear forces of 1 N.

The bump is an important part of the sensor as it transmits the force to the pressure-sensitive layer and protects the sensor. Compared with the other structures, a prismatic structure for the bump enables a more even distribution of the external forces; thus, the structure enhances the sensitivity of the sensor. The top surface dimensions of the prismatic bump affect the efficacy of the force transmission. Therefore, the Ansys Workbench 2023 R1 was used to conduct finite element analysis on prismatic sensors of different sizes. The bottom edge length of the bump was set at 10 mm, while the top edge length ranged from 6 to 9 mm, and the height was 2 mm. A fixed support constraint was applied to the bottom surface of the piezoresistive film, and a positive force of 1 N was applied to the top surface of the bump. The simulation results are shown in Figure 2.

It can be seen from Figure 2a that the bumps with four different edge lengths produce different deformations. The maximum deformation region of the sensor expands from the center toward the edges as the top edge length increases. Furthermore, the effect of the force transmitted to the piezoresistive film is different in the four cases. As shown in Figure 2b, the maximum equivalent stress transmitted to the film via the bumps with edge lengths of 6, 7, 8, and 9 mm is 0.0580, 0.035, 0.042, and 0.0589 MPa, respectively. The regions of larger equivalent stress for the bumps with edge lengths of 6 and 9 mm are mainly distributed at the center and edges of the film, respectively, which prevents the formation of contacts between the piezoresistive film and the electrodes. In comparison, the equivalent force for the 7- and 8- mm bumps is smaller, but they can transmit the force to the pressure-sensitive material layer more uniformly, which facilitates a consistent response of the sensing units and thus improves the accuracy of the measurement. In addition, we analyzed the sensor by applying both normal and shear forces with a magnitude of 1 N, and the equivalent stress of the piezoresistive film is shown in Figure 2c. Due to the resistance variation in the sensor being primarily determined via the contact area between the piezoresistive material and the electrodes, for bump edge lengths of 8 and 9 mm, a larger equivalent stress is distributed over a smaller area of the film, which is not conducive to a reduction in resistance. Similarly, although a bump with an edge length of 6 mm produces a larger equivalent stress and area, the equivalent stress is concentrated in the central region under the action of a single normal force. Overall, the bump with an edge length of 7 mm showed better force transfer under both normal and shear forces.

#### 3.2.2. Layered Structure of the Sensor

**Figure 3:**
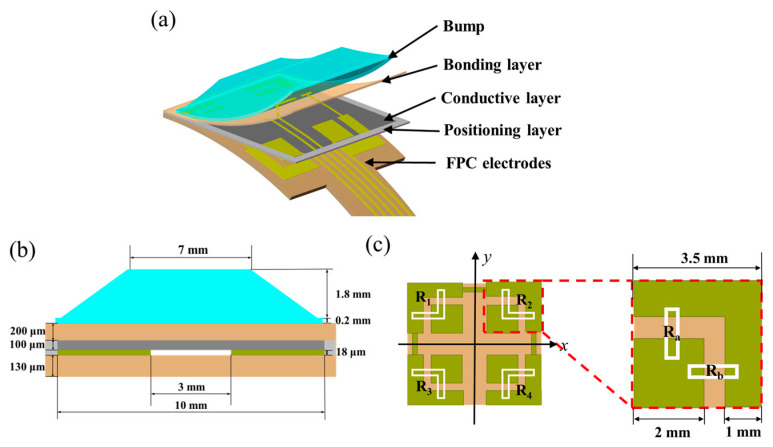
Schematic of the structure of flexible 3D force sensor: (**a**) structure of sensor; (**b**) sensor dimensions; (**c**) patterned electrodes.

As shown in Figure 3a, the structure of the flexible 3D force sensor consists of five layers, namely, a polyimide (PI) sensing film with electrodes, a positioning layer, a conductive layer, a bonding layer, and a surface layer with the bump. The sensor is made of a PI film and PDMS with excellent thermal and chemical stability as well as good mechanical durability. The flexible electrode substrate is made of a double-layer PI with a total thickness of 130 μm, which ensures the flexibility and durability of the sensor; the dimensions of the sensor are shown in Figure 3b. To enable 3D force measurements, the electrodes are patterned as shown in Figure 3c. The electrode structure consists of four independent electrodes at the center and a common ground electrode at the periphery. This arrangement simplifies the overall structure of the tactile sensor, reduces the coupling effects between the sensing units, decreases the manufacturing complexity, and results in a smaller and more compact sensor.

### 3.3. Fabrication Processes

**Figure 4:**
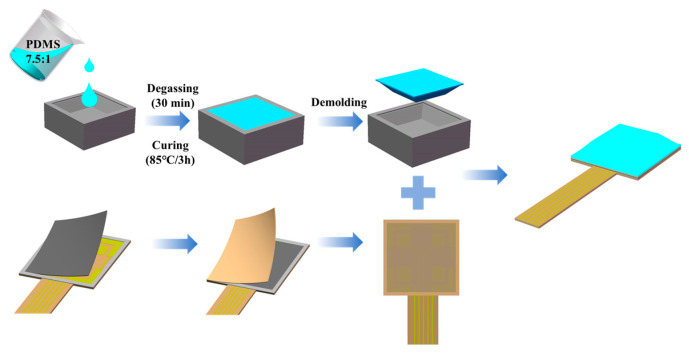
Schematic of the fabrication process of flexible 3D force sensor.

The fabrication process of the sensor is shown in Figure 4. First, a PDMS-to-curing-agent mass ratio of 7.5:1 was added to a measuring cup; such a mass ratio endows the sensor with good elasticity and response. Then, the mixture was stirred manually with a glass rod for 5 min until it was well mixed. The mixture was poured into an aluminum mold and spin-coated at 300 rpm for 3 min. Next, the mold with the mixture was placed in a vacuum dryer for 30 min for evacuation to remove air. The excess mixture above the mold was extruded using a glass plate; subsequently, the mold was placed again in the vacuum dryer at a temperature of 85 °C and heated for 3 h before being demolded. When attaching the flexible printed circuit (FPC) electrodes to the piezoresistive film, a positioning layer was applied in advance around the patterned electrodes to achieve a good fit between the piezoresistive film and the electrodes.

A PET adhesive was applied on top of the piezoresistive film. Due to the surface of the solid material being microscopically rough, the piezoresistive film and the electrodes are not under tension when not loaded, so the conductive network between them is insufficient to provide a good electron transmission (the circuit is open). When a load is applied, the contact area between the piezoresistive material and the electrodes increases, and many conductive pathways are formed, which results in an increase in sensor sensitivity. Finally, the bump and the PET adhesive were bonded using silicone (Cemedine 8008, SongTech, Dongguan, China), and a 200 g weight was placed on top of the bump and left for 24 h for the silicone to cure.

### 3.4. Experimental Setup

**Figure 5:**
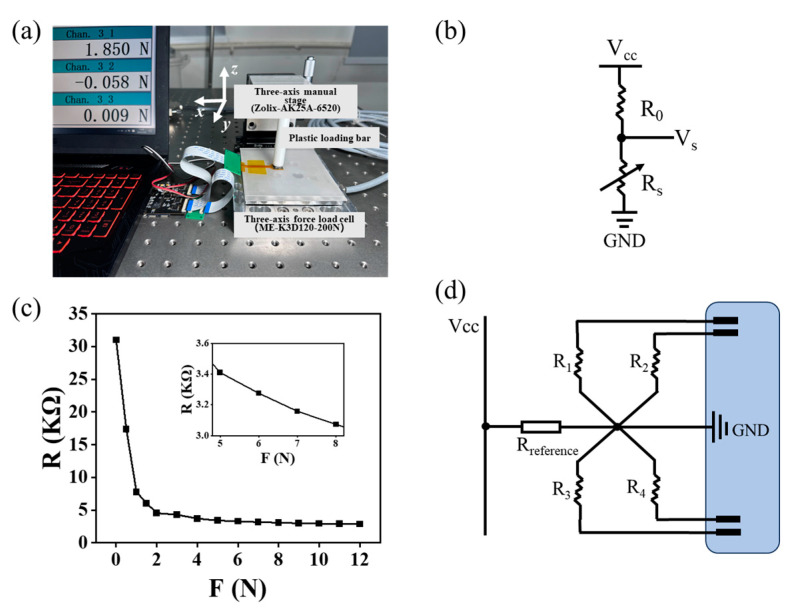
Experimental setup: (**a**) measurement setup of flexible 3D force sensor; (**b**) conventional measurement circuit; (**c**) resistance variation in the sensor at normal force of 0–12 N; (**d**) measurement circuit of the 3D force sensor.

Figure 5a shows the experimental setup used for testing the performance of the sensor, which consisted of three single-axis manual stages (Zolix-AK25A-6520, Zolix, Beijing, China) and a commercial three-axis force sensor (MEK3D120, ME-measurement systems GmbH, Hennigsdorf, Germany). The commercial 3D force sensor has a resolution of up to 1 mN, a measurement range of 0–100 N, and a relative linearity error as low as ±0.2% FS. The loading bar was 3D-printed using a white resin material (Nova, bena5) and mounted on a three-axis manual stage so that the center of the bar was precisely aligned with the sensor. When the loading bar moves in the *x*-, *y*-, and *z*-directions, normal and shear forces are exerted on the sensor. As shown in Figure 5b, during signal acquisition by the sensor, it is common to connect a voltage-divider resistor in series in the circuit. The value of this voltage-divider resistance can be determined based on the resistance variation in the conductive material within the effective working range. The selection of the reference resistor affects the resolution of the sensor output signal, taking the output voltage as an example. The resistance variation range of the conductive material in the effective working range is *R*_min_–*R*_max_, and the output voltage can be expressed with the following equations:

(1) $$V_{\min}=\frac{R_{\min}\cdot V_{cc}}{R_{0}+R_{\min}}\;\mathrm{and}\;V_{\max}=\frac{R_{\max}\cdot V_{cc}}{R_{0}+R_{\max}}$$

(2) $$\;\frac{V_{\max}-V_{\min}}{V_{cc}}=\frac{R_{\max}-R_{\min}}{(R_{0}+R_{\max})\cdot (R_{0}+R_{\min})/R_{0}}$$

where *V*_min_ is the voltage at maximum load; *V*_max_ is the voltage at minimum load; *R*_0_ is the resistance of the reference resistor; *R*_min_ is the resistance at maximum load; *R*_max_ is the resistance at minimum load; and *V*_cc_ is the power supply voltage. From Equation (2), it can be concluded that the amplitude of the output voltage of the sensor is at a maximum when *R*_0_ = (*R_min_*×*R_max_*)^1/2^. According to Figure 5c, the maximum and minimum resistances of the piezoresistive material in the normal operating range are approximately 31 and 3 kΩ, respectively. Therefore, the reference resistor *R*_0_ is determined to be 10 kΩ. To simplify the electrode wiring, the four sensing units of the flexible 3D force sensor were connected in series with the same reference resistor, where each sensing unit corresponds to the variable resistors *R*_1_–*R*_4_ shown in Figure 5d. An STM32F103C8T6 microcontroller was used for data acquisition, equipped with 10 built-in 12-bit ADC converters. The sampling frequency was set to 50 Hz using multiplexed switching sequential-scanning pins to improve the measurement efficiency.

## 4. Results and Discussion

### 4.1. Dynamic Characteristics

**Figure 6:**
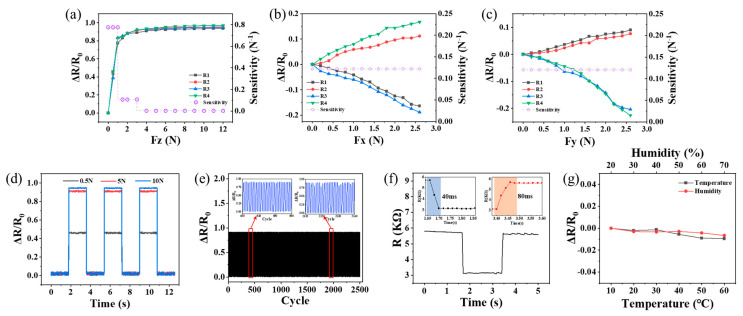
Performance of a unit sensor: (**a**) normal force applied in the *z*-axis; (**b**) shear force applied in the *x*-axis; (**c**) shear force applied in the *y*-axis; (**d**) repeatability of the sensor under different normal forces; (**e**) durability test of the sensor; (**f**) dynamic response and recovery of the sensor under a step of normal force; (**g**) performing resistance tests at different temperatures and humidities.

The sensitivity of the piezoresistive sensor can be expressed as S = (Δ*R*/*R*_0_)/Δ*F*, where Δ*R* is the change in resistance, *R*_0_ is the resistance when no external force is applied, and ΔF is the change in force. Figure 6a shows the rate at which the relative resistance change in the four sensor units varies under a normal force ranging from 0 to 12 N as well as the sensitivity within different pressure ranges. When the pressure is below 1 N, the sensitivity is as high as 0.775 N^−1^, while in the pressure ranges of 1–3 N and 3–12 N, the sensitivity decreases to 0.107 and 0.003 N^−1^, respectively. The results indicate that the sensitivity of the sensor decreases with an increasing external force. Under a low pressure, a significant number of conductive paths are formed between the electrodes and the piezoresistive material, which leads to a rapid decrease in the interface contact resistance. As the pressure gradually increases, the resistance decreases until it reaches a saturated state, and it is difficult to form new conductive networks, which results in a decreased sensitivity of the sensor. Typically, the shear force acting on a sensor is smaller than the normal force, and in most cases, both forces are present simultaneously. The sensitivity of the sensor to the shear force under a normal pressure of 5 N was measured. As shown in Figure 6b,c, as the shear force increases, the change in resistance of the compressed sensing unit becomes less than the change in resistance of the stretched sensing unit. The average sensitivity of the sensor for the *x*- and *y*-axis in the range of 0–2.6 N is 0.122 and 0.12 N^−1^, respectively. In addition, by subjecting the sensor to three consecutive loading and unloading cycles at different normal pressures (0.5, 5, and 10 N; as shown in Figure 6d), stable output signals are obtained, indicating that the proposed sensor can operate under various pressure regimes with sufficient repeatability. To verify the good cyclic performance of the sensor, the sensor was subjected to 2500 compression–release cycles at a normal pressure of 6 N, and the dynamic variation in the relative resistance is shown in Figure 6e. The sensor exhibits good repeatability, stability, and consistency. Furthermore, Figure 6f displays the resistance change in the sensor under rapid loading and unloading of a 5 N normal pressure. Considering the signal change to 90% of the total loading change range as the response time and the signal change to 10% of the total unloading change range as the recovery time, the response and recovery times are found to be 40 and 80 ms, respectively. This proves that the sensor can maintain its good stability in a complex and changeable environment. Figure 6g depicts the influence of temperature and humidity on the sensor-resistance measurement. It implies that the relative resistance of the sensor slightly changes in the range of 10–60 °C and a humidity level within 20–70%, with a maximum difference of 0.94% and 0.66%, respectively. The dynamic test results above indicate that the flexible 3D force sensor designed in this study exhibits an excellent sensing performance.

### 4.2. Calibration and Measurement of Three-Dimensional Force

**Figure 7:**
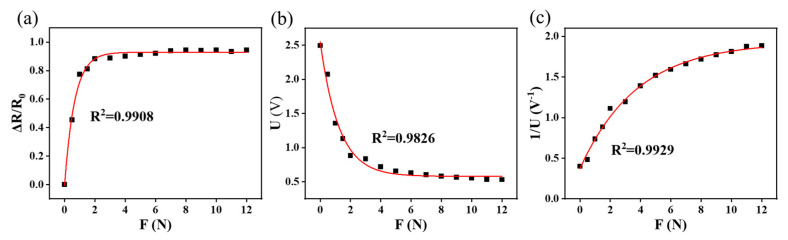
Relationship between the force and different output signals: (**a**) rate of resistance change; (**b**) voltage; (**c**) reciprocal of the voltage.

According to the dynamic test results of the sensor, the output signal of the sensor exhibits a nonlinear relationship with external forces. As shown in Figure 7, the relationships between the three variables (relative rate of change in resistance, voltage, and reciprocal of voltage (U^−1^)) and the pressure were fitted by the least-squares method, and the *R*^2^ values for the three fittings are 0.9908, 0.9826, and 0.9929, respectively. The curve of the force as a function of U^−1^ is the one that could be best fitted. Therefore, the force as a function of U^−1^ was selected as the decoupling model. The decoupling was achieved by combining the reciprocals of the voltages of the four sensing units ($U_{1}^{–1}$, $U_{2}^{–1}$, $U_{3}^{–1}$, and $U_{4}^{–1}$). The decoupling expression is as follows:

(3) $$\left\{ \begin{array}{l} U_{z}^{-1}=\frac{U_{1}^{-1}+U_{2}^{-1}+U_{3}^{-1}+U_{4}^{-1}}{4}\\ U_{x}^{-1}=\frac{\left|U_{2}^{-1}+U_{4}^{-1}-U_{1}^{-1}-U_{3}^{-1}\right|}{2}\\ U_{y}^{-1}=\frac{\left|U_{1}^{-1}+U_{2}^{-1}-U_{3}^{-1}-U_{4}^{-1}\right|}{2} \end{array}\right.$$

**Figure 8:**
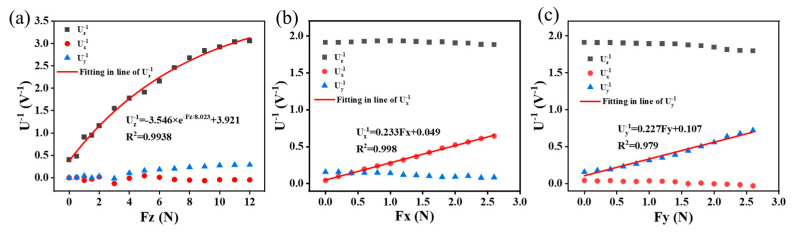
Calibration and test curves of 3D force: (**a**) *z*-axis; (**b**) *x*-axis; (**c**) *y*-axis.

As shown in Figure 8, the relationship between the reciprocals of the voltages ($U_{z}^{–1}$, $U_{x}^{–1}$, and $U_{y}^{–1}$) and the applied force was obtained according to Equation (3). When the force is applied in the z-direction, the output signal increases nonlinearly with increasing force, but the output in the *x*- and *y*-directions is basically unchanged. Similarly, when a force is applied in the *x*- or *y*-direction, the output in the *z*-direction and *y*- or *x*-direction is essentially unchanged. The output signal as a function of the force was fitted in the three directions, and the fitting curves have high *R*^2^ values, that is 0.994, 0.998, and 0.979, respectively. It can be concluded that the reciprocal of the voltage in a given direction increases with the increase in the force in that direction, while the reciprocals of the voltages in the other directions remain basically unchanged. This shows that the sensor has good 3D force-resolution capabilities and avoids crosstalk between the three directions. The relationships between the *F* and U^−1^ components are expressed with the following polynomial functions:

(4) $$\left\{ \begin{array}{l} F_{z}=-8.023\times \ln(|(U_{z}^{-1}-3.92)/3.546|)\\ F_{x}=4.292U_{x}^{-1}-0.210\\ F_{y}=4.405U_{y}^{-1}-0.471 \end{array}\right.$$

### 4.3. Glove-like Sensor Array for Sensing Contact Force

**Figure 9:**
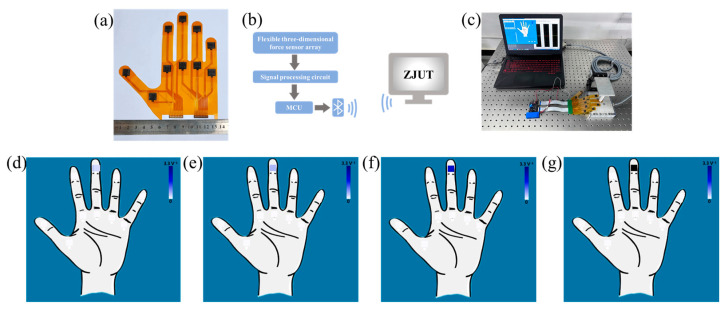
Detection systems and normal force testing: (**a**) tactile glove sensing array; (**b**) schematic diagram of testing system; (**c**) data acquisition system for the tactile glove sensing array; (**d**) 0.2 N normal force display; (**e**) 2 N normal force display; (**f**) 5 N normal force display; (**g**) 12 N normal force display.

The human hand can recognize objects of different sizes, shapes, and materials through receptors in the skin. We prepared a tactile glove using 10 flexible 3D force tactile sensors placed on the five fingertips and the palm, as shown in Figure 9a. Additionally, as shown in Figure 9b,c, a Bluetooth transmission system was established where the sensor array transmits digital signals to the micro-control unit (MCU), which then interfaces with the PC for visualization. Python 3.11 was used to write a visual interface, which can intuitively display the force magnitude and position of the sensing units. The PC interface displays the shape of a hand, and the 10 squares represent 3D force-sensing units. The squares are all white when no force is applied to the sensor array; as the pressure increases, the square color blocks will change from white to blue and finally to black. As shown in Figure 9d–g, when a normal force of 0.5, 2, 5, and 12 N was applied at the middle finger position of the glove sensing array, the reciprocal average voltage of the four sensing units of the single flexible 3D force sensor are 0.451, 1.157, 1.973, and 3.127 V^−1^, which correspond to voltage values of 2.217, 0.864, 0.507, and 0.320 V, respectively. The squares showed as light gray, light blue, blue, and black.

**Figure 10:**
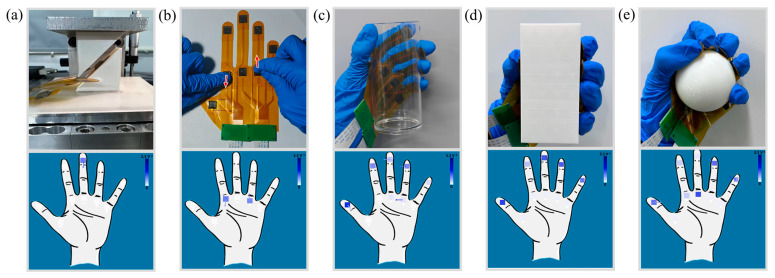
Testing of tactile gloves: (**a**) setup used to apply a 45° force; (**b**) twisting; (**c**) grasping a cylindrical object; (**d**) gripping a square object; (**e**) grabbing a spherical object.

In addition, the interface displays information about the shear force. As shown in Figure 10a, a 45° spatial force was applied to the sensor by two 45° inclined tables. The sensor was placed on the inclined plane of one of the inclined tables, and the inclined plane of the other inclined table was parallel to the bump of the sensor. In this case, the normal and shear forces are equal. The arrows show the direction of the shear force, and the squares show the normal force in the interface; they are of the same color. The sensor array was tested for twisting, as shown in Figure 10b; the direction of the shear force displayed on the 3D force display interface was the same as the direction of the real twist.

To investigate the force distribution during object grasping, experiments on gripping were conducted on the sensor array. When gripping a cylindrical acrylic cup, the force exerted by the hand is mainly concentrated at the thumb, index finger, and middle finger, while the sensors on the little finger and palm only touch the cup wall without generating a significant force; this explains the lighter colors of the corresponding sensors (Figure 10c). When grasping a rectangular object, only the fingertips of the five fingers are in contact with the object, while the middle palm portion is uncontacted. The corresponding fingertip sensors in the interface show different magnitudes of blue, while the uncontacted sensors in the palm show no color (Figure 10d). The force of the hand is mainly concentrated on the fingertip sensors and the palm part of the sensors when grasping a spherical object. As shown in Figure 10e, the sensors in the middle and index fingers as well as the palm have good contact with the sphere. However, the curved surface of the sphere results in the bumps of the sensors for the thumb, little finger, and ring finger not being able to make complete contact with the sphere. Through the pre-calibration of the flexible 3D force sensors, the direction of the force applied to the ring finger and little finger is the upper left side. In addition, due to the small radius of the sphere, the two sensors at the point where the ring finger and the little thumb are connected to the palm did not make contact with the surface of the sphere, so there is no corresponding color display at this position, which is also consistent with the actual grasping of the sphere.

Through the display of the glove array, a more intuitive reflection of the force situation during object grasping can be achieved, and the 3D force information can provide more accurate and comprehensive force data. These sensors could be used in robots to grasp and identify the shape, size, and other characteristics of objects.

## 5. Conclusions

In summary, we developed a sandwich-structured flexible 3D force tactile sensor using a simple and efficient mold method and a layer-by-layer assembly approach. This sensor consisted of four force-sensing units under a bump structure and included a positioning layer to ensure a good match between the piezoresistive material and the electrodes. Additionally, finite element analysis was conducted on the bump at the top of the sensor. The effectiveness of force transmission for bump surfaces with different side lengths on the top was evaluated through the application of normal and shear forces to the bump surface to simulate actual force conditions. The bump with a side length of 7 mm was the most effective at transmitting the force. The sensor can be used to measure normal forces in the range of 0–12 N and shear forces up to 2.6 N. The sensor output remained stable over 2500 loading/unloading cycles, demonstrating excellent repeatability. Specifically, the sensor exhibited a high sensitivity (0.775 N^−1^, equivalent to 37.9 Pa^−1^ in the range of 0−27.8 kPa) for normal pressures ranging from 0 to 1 N, and a sensitivity of 0.122 N^−1^ for shear forces up to 2.6 N, with rapid response and recovery times (40 and 80 ms, respectively). An experimental platform and a testing circuit were established for the detection of 3D forces, and the sensor was calibrated in three directions. Fitting the force and output signals enabled the development of a 3D force-decoupling model, permitting the accurate determination of the magnitude and direction of 3D forces based on the fitting curves. Additionally, we prepared a glove-like sensor array and designed it to be able to visualize the real-time force applied to the sensors when the glove was grasping an object. These sensors are promising for numerous applications, such as agile robotic operations, electronic skins, and human–machine interaction technology.

## Figures and Tables

**Table 1:** Material properties of velostat.

Volume Resistivity Ω·cm	Surface Resistivity kΩ/cm^2^	Operating Temperature Range °C	Thickness μm
≤500	≤31	−45–65	100

## Data Availability

The original contributions presented in the study are included in the article, further inquiries can be directed to the corresponding author.
